# Using Real-Time Social Media Technologies to Monitor Levels of Perceived Stress and Emotional State in College Students: A Web-Based Questionnaire Study

**DOI:** 10.2196/mental.5626

**Published:** 2017-01-10

**Authors:** Sam Liu, Miaoqi Zhu, Dong Jin Yu, Alexander Rasin, Sean D Young

**Affiliations:** ^1^ Institute for Prediction Technology Department of Family Medicine University of California Los Angeles, CA United States; ^2^ Department of Computer Science University of California Los Angeles, CA United States; ^3^ College of Computing and Digital Media DePaul University Chicago, IL United States

**Keywords:** social media, twitter messaging, stress, monitoring

## Abstract

**Background:**

College can be stressful for many freshmen as they cope with a variety of stressors. Excess stress can negatively affect both psychological and physical health. Thus, there is a need to find innovative and cost-effective strategies to help identify students experiencing high levels of stress to receive appropriate treatment. Social media use has been rapidly growing, and recent studies have reported that data from these technologies can be used for public health surveillance. Currently, no studies have examined whether Twitter data can be used to monitor stress level and emotional state among college students.

**Objective:**

The primary objective of our study was to investigate whether students’ perceived levels of stress were associated with the sentiment and emotions of their tweets. The secondary objective was to explore whether students’ emotional state was associated with the sentiment and emotions of their tweets.

**Methods:**

We recruited 181 first-year freshman students aged 18-20 years at University of California, Los Angeles. All participants were asked to complete a questionnaire that assessed their demographic characteristics, levels of stress, and emotional state for the last 7 days. All questionnaires were completed within a 48-hour period. All tweets posted by the participants from that week (November 2 to 8, 2015) were mined and manually categorized based on their sentiment (positive, negative, neutral) and emotion (anger, fear, love, happiness) expressed. Ordinal regressions were used to assess whether weekly levels of stress and emotional states were associated with the percentage of positive, neutral, negative, anger, fear, love, or happiness tweets.

**Results:**

A total of 121 participants completed the survey and were included in our analysis. A total of 1879 tweets were analyzed. A higher level of weekly stress was significantly associated with a greater percentage of negative sentiment tweets (beta=1.7, SE 0.7; *P*=.02) and tweets containing emotions of fear (beta=2.4, SE 0.9; *P*=.01) and love (beta=3.6, SE 1.4; *P*=.01). A greater level of anger was negatively associated with the percentage of positive sentiment (beta=–1.6, SE 0.8; *P*=.05) and tweets related to the emotions of happiness (beta=–2.2, SE 0.9; *P*=.02). A greater level of fear was positively associated with the percentage of negative sentiment (beta=1.67, SE 0.7; *P*=.01), particularly a greater proportion of tweets related to the emotion of fear (beta=2.4, SE 0.8; *P*=.01). Participants who reported a greater level of love showed a smaller percentage of negative sentiment tweets (beta=–1.3, SE 0.7; *P*=0.05). Emotions of happiness were positively associated with the percentage of tweets related to the emotion of happiness (beta=–1.8, SE 0.8; *P*=.02) and negatively associated with percentage of negative sentiment tweets (beta=–1.7, SE 0.7; *P*=.02) and tweets related to the emotion of fear (beta=–2.8, SE 0.8; *P*=.01).

**Conclusions:**

Sentiment and emotions expressed in the tweets have the potential to provide real-time monitoring of stress level and emotional well-being in college students.

## Introduction

### Background

College can be stressful for many freshmen as they cope with a variety of academic, personal, and social pressures [1]. Although not all stress is negative, a certain level of stress can be beneficial to help improve performance. However, too much stress can adversely affect health [2]. In the annual survey of the American Freshman, the number of students reported feeling overwhelmed and stressed has increased steadily in the last decade [3]. Over 50% of college students suffer significant levels of stress during a typical college semester [4].

High levels of stress is known to negatively impact a person’s emotional well-being by increasing the degree of negative emotions (eg, anger, fear) and decreasing the levels of positive emotions (eg, love, happiness) [5]. Increased levels of stressful life events have been shown to be associated with anxiety, depression, and suicidal ideation in college students [6-8]. Excess stress can also negatively affect physical health, such as increased sleep disturbances, decreased immune function, and loss of appetite [9-11]. Consequently, there is a need to find innovative and cost-effective strategies to help identify those students experiencing high levels of stress and negative emotions early on so that they can receive the appropriate treatment in order to prevent future mental illnesses [4].

Social media use, such as Twitter and Facebook, has been rapidly growing, and research has already shown that data from these technologies can be used for novel approaches to public health surveillance [12-15]. Twitter usage among young adults has increased 16% from 2012 to 2014. Currently, 32% of adults of the ages 18-29 years use Twitter, and the usage is expected to increase steadily in the future [12-15].

People often have the need to share their emotions and experiences [16]. Researchers have theorized that emotional sharing may fulfill a socio-affective need by eliciting attention, affection, and social support. Consequently, this may help individuals cope with their emotions and provide an immediate relief [16]. Users often share their thoughts, feelings, and opinions on these social media platforms, and as a result, social media data may be used to provide real-time monitoring of stress and emotional state among college students [17]. Previous studies have shown that Twitter data can be used to monitor a wide range of health outcomes, such as detecting human immunodeficiency virus infection outbreaks and predicting an individual’s risk of depression [12,18]. For example, De Choudhury et al conducted one of the first studies that used an individual’s tweets to predict the risk of depression [18]. The authors found that certain features extracted from a person’s tweets collected over a 1-year period were highly associated with the risk of depression in adults, such as raised negative sentiment in the tweets, frequent mentions of antidepressant medication, and greater expression of religious involvement. Currently, no studies have examined whether Twitter data can be used to monitor stress level and emotional state among college students. Studying this topic is important because the large amount of social media data from college students’ frequent use of social media can be used to help university officials and researchers monitor and reduce stress among college students [19].

### Objectives

The primary objective of our study, therefore, was to investigate whether students’ perceived levels of stress were associated with the sentiment and emotions of their tweets. The secondary objective was to explore whether students’ emotional state was associated with the sentiment and emotions of their tweets. Because of the high level of stress among incoming freshman students, we decided to study this population in their first quarter of school. We hypothesized that higher levels of stress, anger, and fear would be associated with a greater proportion of tweets with negative sentiment and emotions (anger and fear). Conversely, we hypothesized that higher levels of love and happiness would be associated with a greater proportion of tweets with positive sentiment and emotions (love and happiness).

## Methods

### Overview

This was a longitudinal observational study that took place during the fall semester of 2015. A total of 181 first-year undergraduate freshman students at University of California, Los Angeles (UCLA) were recruited. In order to be eligible for the study, participants were required to be freshmen, have a Twitter account, post at least two tweets per week, and be willing to share their Twitter handle. Data collected between November 2 and November 8, 2015, were extracted for analysis. Ethics approval was obtained from the UCLA Research Ethics Board.

### Recruitment and Study Protocol

Participants were informed about the study through flyers on social media websites and on UCLA campus between September 14 and October 12, 2015. Participants who provided consent were asked to complete a Web-based questionnaire that assessed their levels of stress and emotional state for the last 7 days. Psychometric measures of stress were constructed to assess the extent to which individuals find their lives to be overloaded. Participants were to identify their overall levels of stress and sources of stress for the last 7 days on a 1-5 Likert scale (1=not at all stress, 2=low stress, 3=average stress, 4=high stress, 5=extremely high stress). Previous research has identified basic human emotions, which included feelings of anger, fear, love, and happiness [20,21]. Participants were asked to rate their emotional state (feelings of anger, fear, love, and happiness) for the last 7 days on a 1-5 Likert scale (1=extremely low, 2=low, 3=somewhat, 4=strong, 5=extremely strong). All questionnaires were completed within a 48-hour period. Demographic information was collected, including age, gender, ethnicity, and students’ area of study. All tweets posted by the participants from that week (November 2 to 8, 2015) were mined and manually categorized based on their sentiment (positive, negative, neutral) and emotion (anger, fear, love, happiness) expressed. Participants were rewarded with a US $5 gift card for completing the survey.

### Twitter Sentiment and Emotion Analysis

All participants’ tweets were extracted using a Twitter streaming application programming interface. To ensure the accuracy of the coding, a random subset of the tweets (n=100) was selected first. These tweets were then coded independently by 2 domain experts (SL and SY) based on the tweets’ sentiment and emotions. The interrater reliability was .83. In the event of conflicting opinions, resolution was achieved by consensus. The domain expert (SL) then manually coded the remainder of the tweets.

The tweets were first coded as expressing positive, negative, and neutral sentiments. To ensure the quality of the coding, a publicly available set of labeled tweets (n=150) by workers on Amazon’s Mechanical Turk was used as a reference prior to sentiment labeling [22]. The tweets were then categorized into 4 primary emotions of anger, fear, love, and happiness in order to reflect the emotional state that the students felt during the week. Based on previous work by Ekman’s list of basic emotions [21] and Parrots’ classification of emotions [23], we created a coding scheme to categorize other emotions that led to or could be included in the 4 primary emotions of anger, fear, love, and happiness. For example, (1) emotion of love also included emotions of affection, kindness, passion, and longing; (2) emotion of happiness also included emotions of joy, cheerfulness, and excitement; (3) emotion of anger also included emotions of disgust, torment, and judgment; (4) emotion of fear also included emotions of nervousness, stress, and sadness. Ambiguous tweets that could not be understood were labeled as unknown. Retweets were excluded from the analysis, as it was a challenge to interpret the sentiment and emotions. For example, it was unclear whether the participants were retweeting because they shared the sentiment of the person who originally tweeted it or they were retweeting for support toward that person.

### Statistical Analysis

Descriptive statistics were used to summarize baseline demographic characteristics, levels of stress, emotional well-being, and the number of positive, neutral, negative, anger, fear, love, and happiness tweets. Ordinal regressions were used to assess whether weekly levels of stress and emotional states (anger, fear, love, happiness) were associated with the percentage of positive, neutral, negative, anger, fear, love, or happiness tweets. Due to the small number of participants who scored either 1 (extremely low) or 5 (extremely high) on the Likert scales for weekly levels of stress and emotional well-being, it is common to combine the cells [24]. We combined scores of 1 (extremely low) with 2 (low) and 5 (extremely high) with 4 (high). As result, the dependent variable in the ordinal regression for self-report levels of stress and emotional state contained 3 levels (low, average, and high). In order to account for the differences in the number of tweets people posted, we calculated the percentage of tweets expressing positive, negative, and neutral sentiments, and emotions of anger, fear, love, and happiness to be included in the regression analyses. Based on previous research, all models were adjusted for covariates including age, ethnicity, and gender [25-28]. In assessing overall model fit, the goodness-of-fit measure (–2 log likelihood) was used. Smaller values of the –2 log likelihood measure indicated better model fit [29]. Data were analyzed using SPSS version 21 (IBM Corp). Data were reported as mean (SD), and statistical significance was assumed at *P*≤.05.

## Results

### Participants

A total of 121 participants completed the survey and were included in our analysis. Baseline participant characteristics are presented in Table 1. The prevalence of white and Asian ethnic groups in this study were underrepresented, whereas African American and Latino ethnic groups were overrepresented in the distribution in UCLA [30]. This may be due to the popularity of Twitter usage among African Americans and Latinos [19].

### Stress Level and Emotional State

The distribution of stress level and emotional state is displayed in Table 2. The majority of participants (~80%) reported average or high levels of stress. The most commonly reported stressors by students were related to attending class, completing homework, and dealing with self-image. Overall, the majority of participants (~50%-70%) reported lower to average levels of anger and fear, and average to higher levels of love and happiness. Stress was significantly correlated with anger (*r*=.17, *P*=.05) and fear (*r*=.51, *P*<.001). Emotions of anger were also positively correlated with fear (*r*=.41, *P*<.001). Meanwhile, emotions of happiness were significantly correlated with love (*r*=.52, *P*<.001).

**Table 1:** Baseline characteristics.

Characteristic (N=121)		Mean (SD) or n (%)
Age		18.14 (SD 0.49)
**Gender**		
	Female	76 (62.8%)
**Ethnicity**		
	White	25 (20.7%)
	African American	13 (10.7%)
	Latino	35 (28.9%)
	Asian	33 (27.3%)
	Other	15 (12.4%)
**Areas of study**		
	Health science	55 (45.4%)
	Business	10 (8.3%)
	Math or engineering	15 (12.4%)
	Social science or arts	24 (19.8%)
	Undeclared	17 (14.0%)

**Table 2:** Summary levels of stress and emotions.

Stress levels and emotions	Low, n (%)	Average, n (%)	High, n (%)
Overall stress	26 (21.5%)	48 (39.7%)	47 (38.8%)
Emotions of anger	68 (56.1%)	32 (26.4%)	21 (17.4%)
Emotions of fear	61 (50.4%)	31 (25.6%)	29 (24.0%)
Emotions of love	38 (31.4%)	34 (28.1%)	49 (40.5%)
Emotions of happiness	20 (16.5%)	38 (31.4%)	63 (52.1%)

### Sentiment and Emotional Analysis of the Tweets

A total of 1879 tweets were included in our analysis. Out of these tweets, there were 490 (26.08%) positive sentiment tweets, 410 (21.82%) negative sentiment tweets, 590 (31.40%) neutral tweets, and 389 (20.70%) unknown tweets. Of the positive sentiment tweets, 201 tweets expressed emotions of love, kindness, support, inspiration, or longing, for example, “Blessed At The End Of Every Day”; “Lord do I have such amazing and supportive friends”; and 289 tweets expressed emotions of happiness, joy, or excitement, for example, “Finally done wit midterms”; “I just ate next to Zaza Pachulia at In N Out’s westwood!!!” Of the negative sentiment tweets, 124 tweets expressed emotions of anger, insult, dogma, or judgment, for example, “I swear these people just stare at me it annoying”; “I hate essays with a passion *…* ”; and 286 tweets expressed emotions of either fear, stress, or sadness, for example, “College is just too stressful”; “I chose Netflix and sleep over studying and now 1 hour before my test I hate myself.” The participants posted on average 14 (SD 23, range 2-144) tweets during the 7-day period. The average number of tweets per person that contain positive, negative, and neutral sentiments were 3.7 (SD 3.4, range 0-35), 3.1 (SD 5.9, range 0-43), and 4.5 (SD 8.2, range 0-52), respectively. The mean number of tweets per person containing emotion of anger was 1.1 (SD 1.9, range 0-10), and the mean number of tweets containing emotion of fear was 2.2 (SD 4.3, range 0-33).

The relationship between individuals’ stress level, emotional state, and sentiment and emotions expressed in tweets is shown in Table 3. In our ordinal regression analysis, we found that a higher level of weekly stress was significantly associated with a greater percentage of negative sentiment tweets, tweets containing fear, and tweets containing love. The best-fitting model for predicting weekly levels of stress was using tweets related to the emotions of fear (–2 log likelihood=181.3; χ^2^_7_=21.2; *P*=.004). A greater level of anger was negatively associated with the percentage of positive sentiment and tweets related to the emotions of happiness. The best-fitting model for predicting weekly levels of anger was using tweets related to the emotions of happiness (–2 log likelihood=161.3; χ^2^_7_=7.1; *P*=.03). A greater level of fear was positively associated with the percentage of negative sentiments, particularly a greater proportion of tweets containing the emotion of fear. The best-fitting model for predicting weekly levels of fear was using tweets related to the emotions of fear (–2 log likelihood=180.0; χ^2^_7_=18.2; *P*=.01). Participants who reported a greater level of love showed a smaller percentage of negative sentiment tweets (–2 log likelihood=217.8; χ^2^_7_=15.1; *P*=.03). Finally, emotions of happiness were positively associated with the percentage of tweets related to the emotion of happiness and negatively associated with the percentage of negative sentiment tweets and tweets related to the emotion of fear. The best-fitting model for predicting weekly emotions of happiness was using tweets related to the emotions of fear (–2 log likelihood 179.0; χ^2^_7_ 20.1; *P*=.005).

**Table 3:** The relationship between individuals’ stress level, emotional state, and sentiment and emotions expressed in tweets (model adjusted for age, gender, and ethnicity).

Tweet sentiments and emotions	Stress		Anger		Fear		Love		Happiness	
	Beta (SE)	*P*	Beta (SE)	*P*	Beta (SE)	*P*	Beta (SE)	*P*	Beta (SE)	*P*
Percent of negative sentiment	1.72 (0.74)	.02	0.85 (0.70)	.22	1.67 (0.73)	.02	–1.3 (0.70)	.05	–1.69 (0.70)	.02
Percent of positive sentiment	0.10 (0.71)	.89	–1.60 (0.80)	.05	–0.78 (0.72)	.28	0.54 (0.71)	.45	1.24 (0.72)	.09
Percent of neutral sentiment	–0.42 (0.69)	.54	–0.27 (0.72)	.71	0.26 (0.69)	.70	–0.05 (0.70)	.95	–0.92 (0.69)	.18
Percent of tweets related to the emotion of anger	–0.03 (1.06)	.98	0.35 (1.1)	.75	0.19 (1.1)	.86	0.62 (1.1)	.55	1.37 (1.17)	.24
Percent of tweets related to the emotion of fear	2.37 (0.90)	.01	0.90 (0.79)	.25	2.11 (0.83)	.01	–1.42 (0.80)	.07	–2.8 (0.84)	.01
Percent of tweets related to the emotion of love	3.60 (1.44)	.01	0.88 (1.35)	.51	2.55 (1.38)	.06	–0.94 (1.3)	.48	–1.70 (1.31)	.20
Percent of tweets related to the emotion of happiness	–0.92 (0.73)	.21	–2.22 (0.94)	.02	–1.54 (0.80)	.06	0.93 (0.75)	.21	1.79 (0.79)	.02

## Discussion

### Principal Findings

The main finding of this study was that tweet sentiment was associated with participants’ future survey about their emotions and stress. Specifically, higher levels of stress and emotion of fear were associated with a greater percentage of negative sentiments and percentage of tweets related to fear. Meanwhile, emotions of love and happiness were negatively associated with the percentage of negative sentiments and percentage of tweets related to the emotion of fear. Interestingly, perceived level of stress was also positively associated with the percentage of tweets with love and hope. Overall, these findings provide evidence that real-time social media data may be used to monitor the psychological health of college students.

There have been previous studies showing that content of geo-tagged tweets (eg, frequency of keywords) can be extracted to predict disease outbreaks at a population level [12,15,18]. However, there is a lack of studies that have examined whether the content of social media data can be used to monitor psychological health at an individual level. In this study, we have built on previous study methods of analyzing the tweets [12,31]. To our knowledge, this is one of the first studies that (1) categorized the tweets with increased degree of granularity of emotional state (eg, anger, fear, love, happiness) and (2) found that certain emotions expressed in individuals’ tweets were better predictors of stress level and emotional state. An interesting finding in this study, contrary to our hypothesis, was that when individuals experienced a higher level of stress, they were more likely to post tweets related to both fear and love or hope. A possible explanation for this is that individuals may post tweets related to love and hope as a coping mechanism and may find it to be comforting while experiencing a higher levels of stress [18,32].

The results from this study have several research implications. First, these findings have furthered our understanding of the types of information that can be extracted from social media data and used to monitor individuals’ levels of stress and emotional well-being. Second, our results suggest that it may be possible to create a new public health surveillance tool to monitor and predict stress level and emotional state among college students. This new tool can help school administrators implement targeted health interventions for those individuals at risk for high levels of psychological distress. Consequently, this can help improve students’ overall health and enhance their academic experience. Finally, the findings from this study can help create a new area of research, and the methods learned can be applied to other population groups (eg, individuals at risk for heart disease).

### Limitations

A limitation of this study was limited sample size and that only freshman college students were included. It may be possible that participants may change their Web-based behavior when they know they are part of the study (ie, Hawthorne effect). Personality and characteristic differences may also influence the frequency and the types of content posted on social media. Overall these factors may limit the ability to generalize our findings. Another limitation was that the psychometric questionnaires used have not been previously validated. We also excluded retweets and only included a week of tweets in our analysis. It may be possible that individuals may have expressed their levels of stress and their emotional state in their retweets or outside the time frame that the tweets were captured. Thus, future studies need to examine the methods to analyze retweets and the optimal time frame that the tweets need to be captured in order to provide accurate predictions of individuals’ levels of stress and emotional well-being. Furthermore, the tweets in this study were manually categorized into 4 types of emotions. In order to scale tweets both as a cost-effective surveillance method and as a tool for developing insights into individuals’ health, natural language processing and machine-learning techniques need to be developed to accurately label the tweets into various emotional categories. Finally, levels of stress and emotional state were measured at only one time point in this study. Future research needs to examine how observed changes in the measures of emotions extracted from Twitter data predict changes in the levels of stress and emotional well-being. We plan to pursue this in future studies by collecting Twitter data and psychological measures of college students in a longitudinal study.

### Conclusions

The ability to use real-time social media data to provide health surveillance has a significant public health application. The results of this study suggest that sentiment and emotions expressed in the tweets have the potential to provide real-time monitoring of stress level and emotional well-being in college students. Future studies can build on the methods used in this study to further refine the ways of utilizing real-time social media data for monitoring the levels of stress and emotional well-being.

## Abbreviations

UCLA: University of California, Los Angeles
